# Going Beyond Bacteria: Uncovering the Role of Archaeome and Mycobiome in Inflammatory Bowel Disease

**DOI:** 10.3389/fphys.2021.783295

**Published:** 2021-12-06

**Authors:** Yashar Houshyar, Luca Massimino, Luigi Antonio Lamparelli, Silvio Danese, Federica Ungaro

**Affiliations:** ^1^IBD Center, IRCCS Humanitas Research Hospital, Milan, Italy; ^2^Department of Gastroenterology and Digestive Endoscopy, IRCCS Ospedale San Raffaele, Milan, Italy; ^3^Faculty of Medicine, Università Vita-Salute San Raffaele, Milan, Italy

**Keywords:** intestinal microbiota, archaeome, mycobiome, inflammation, inflammatory bowel disease, immunity

## Abstract

Inflammatory Bowel Disease (IBD) is a multifaceted class of relapsing-remitting chronic inflammatory conditions where microbiota dysbiosis plays a key role during its onset and progression. The human microbiota is a rich community of bacteria, viruses, fungi, protists, and archaea, and is an integral part of the body influencing its overall homeostasis. Emerging evidence highlights dysbiosis of the archaeome and mycobiome to influence the overall intestinal microbiota composition in health and disease, including IBD, although they remain some of the least understood components of the gut microbiota. Nonetheless, their ability to directly impact the other commensals, or the host, reasonably makes them important contributors to either the maintenance of the mucosal tissue physiology or to chronic intestinal inflammation development. Therefore, the full understanding of the archaeome and mycobiome dysbiosis during IBD pathogenesis may pave the way to the discovery of novel mechanisms, finally providing innovative therapeutic targets that can soon implement the currently available treatments for IBD patients.

## Introduction

The human gastrointestinal (GI) tract is the host of 10^13^-to-10^14^ microorganisms since birth (Gill et al., 2006) when the GI tract starts being colonized by microbial species, forming the gut microbiota (Barko et al., 2018). Changes in specific microbial species abundances and diversity occur until adulthood when the gut microbiota becomes more stable and in symbiosis with the host (Yatsunenko et al., 2012).

Microbial species composing the gut microbiota include bacteria, protozoa, eukaryotes, fungi, viruses, and archaea (Thursby and Juge, 2017). All these entities interact with each other and with the host, existing in a continuum of predator-prey interactions (Frank et al., 2007) which are pivotal for ensuring human health by participating in a variety of physiological functions, such as regulating host immunity, protection against pathogens, as well as host energy harvesting (Clemente et al., 2012; Zhang et al., 2015).

However, symbiotic relationships between microbiota and host may be disrupted by different factors such as antibiotic treatments, dietary habits, lifestyle, environmental triggers, and pathologic conditions leading to gut dysbiosis (Hasan and Yang, 2019), mainly consisting of a reduction of commensal bacteria and increase of enteric pathogens which may lead to disease condition (Hasan and Yang, 2019), such as Inflammatory Bowel Disease (IBD).

Inflammatory Bowel Disease (IBD) includes two main types of chronic inflammatory conditions, ulcerative colitis (UC) and Crohn’s disease (CD) whose aetiopathogenesis is still unknown (Matsuoka and Kanai, 2015; Presti et al., 2019). Although CD and UC differ in disease location and symptoms (Rodrigues et al., 2020), they are both featured by a resolution-failing mucosal immune response (Strober et al., 2007; Shim, 2013), a dysfunctional gut epithelial barrier that fails to defend against pathogens (Presti et al., 2019), and an intestinal dysbiosis (Shim, 2013; Massimino et al., 2021).

Previous studies revealed that IBD patient intestinal microbiota is characterized by a reduced abundance of *Firmicutes* and *Bacteroidetes*, as compared to the healthy individuals, whereas the proportion of *Proteobacteria* and *Actinobacteria* are increased (Walker et al., 2011; Dong et al., 2019). Moreover, a lower abundance of *Faecalibacterium prausnitzii*, the main butyrate producer in the human intestine (Lopez-Siles et al., 2017), has been reported for IBD patients as compared to the healthy subjects. Indeed, the reduced levels of this commensal causes reduced levels of butyrate, known as a proinflammatory cytokine inhibitor, thus sustaining, chronic intestinal inflammation (Presti et al., 2019). Interestingly, numerous studies have been associating increased numbers of virulent *Escherichia coli* strains in IBD patients compared to healthy controls (Mirsepasi-Lauridsen et al., 2019), especially when focusing on IBD patients during disease relapses (Burke and Axon, 1988; Mirsepasi-Lauridsen et al., 2019).

Along with bacteria, the viral component of the gut microbiota, the enteric virome, has been recently characterized as a large and composite community made of both eukaryotic-targeting and prokaryotic-targeting viruses (bacteriophages) (Yue et al., 2019). While bacteriophages contribute to the maintenance of bacterial composition of the gut microbiota and changes in their abundances (virome dysbiosis) may affect bacteria diversity in the GI tract (Lin and Lin, 2019), the gut eukaryotic viruses (Taboada et al., 2021) may exert beneficial effects and directly impact the host’s cells (Ungaro et al., 2019b). Gut virome dysbiosis was reported also in IBD patients and many studies profiled virome composition in the context of GI disease, as extensively reviewed elsewhere (Lin and Lin, 2019; Ungaro et al., 2019a; Massimino et al., 2021).

Despite the extensive analysis of the bacteriome and the virome, archaeome (archaeal components) and mycome (fungal and yeast component) populating the GI tract have been poorly investigated so far, mainly because of the lack of appropriate and reproducible techniques for their profiling (Ungaro et al., 2019a).

This minireview aims to compile all notions depicting these two neglected components of the intestinal microbiota as, on one hand, possible contributors to the maintenance of the intestinal homeostasis and, on the other, precipitators of IBD, eventually prompting future studies toward their better definition and comprehension as actors within the intestinal microbiota.

## The Gut Archaeome and the Mycobiome: From Tissue Physiology to Inflammatory Bowel Disease Pathogenesis

### The Gut Archaeome

Archaea constitute a domain of single-celled organisms alongside two other domains, eukarya, and bacteria (Woese et al., 1990). Although archaea share some features with both bacteria (lack of nucleus, introns, the presence of a single circular chromosome) and eukaryotes (presence of histones for chromosomal DNA packaging) (Gaci et al., 2014), they are classified as a distinct class of organisms that comprises two major kingdoms: *Euryarchaeota* encompassing the methanogens and their phenotypically diverse relatives, and *Crenarchaeota* comprising the relatively tight clustering of extremely thermophilic archaebacteria, whose general phenotype appears to resemble the ancestral phenotype of the Archaea (Woese et al., 1990).

Archaea have the distinctive feature of colonizing a broad range of habitats, because of their evolutionary advantage in using specific pathways metabolizing a versatile panel of energy sources, ranging from the sunlight to both organic and inorganic substances (Valentine, 2007). Now we know that archaea are not only extremophile species colonizing severe environments, but they can be also found in moderate climates and can populate plants and animal intestines, representing an important constituent of the gut microbiota (Janssen and Kirs, 2008; Berg et al., 2016; Raymann et al., 2017).

Previous pieces of evidence positioned the archaeal species as ranging from 0.1 to 21.3% of the microbial entities colonizing the digestive tract (Kim et al., 2020). Recently, the development of culture-independent methods (i.e., Next-Generation Sequencing) has been opening a new horizon for the study of the composition of gut microbiota, where the methanogens have been suggested as a predominant archaeal group among gut microbial entities (Matijašić et al., 2020) and their colonization rate ranges from 25 to 95% of human stools (Stewart et al., 2006; Dridi et al., 2009; Hoffmann et al., 2013). Specifically, methanogens perform anaerobic respiration generating methane as a final product of metabolism (methanogenesis). Indeed, they decrease the gas pressure in the colon by consuming H_2_ and CO_2_ to produce methane (Gaci et al., 2014). Therefore, archaeal methanogens are likely to compete with sulfate-reducing bacteria for H_2_ production in the human colon (Conway de Macario and Macario, 2009). Consequently, unbalance between methanogens and sulfate-reducing bacteria may alter gut mucosal homeostasis, resulting in intestinal dysbiosis.

**FIGURE 1:**
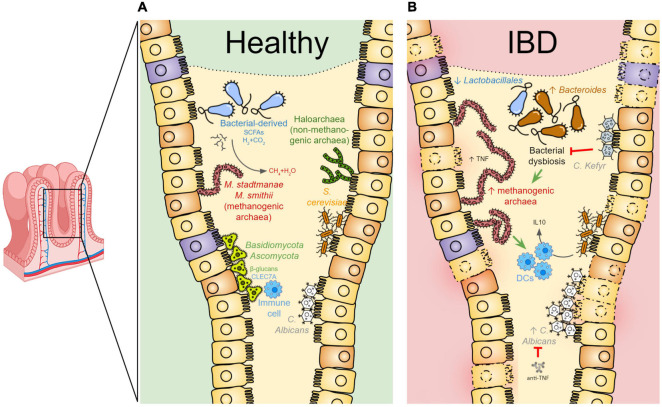
The gut archaeome and mycobiome communities in health and disease. **(A)** In healthy conditions, the intestinal mucosa is colonized by specific archaeal and fungal species contributing, together with the other components of the microbiota, to the overall gut physiology. The archaeal methanogens [i.e., *Methanobrevibacter (M.) smithii* and *Methanosphaera (M.) stadtmanae* species] exist in a syntrophic relationship with bacterial species within the gut. Short-chain fatty acids (SCFAs) as well as hydrogen gas (H_2_), produced by anaerobic bacterial fermentation, are used by methanogens to decrease the gas pressure in the colon by consuming H_2_ and CO_2_ to produce methane. In physiologic conditions, the human gut is also colonized by the haloarchaea. In parallel, the human gut mycobiota is predominantly characterized by *Ascomycota, Basidiomycota, Candida (C.) albicans*, and *Saccharomyces (S.) cerevisiae.* Fungi can be detected by the host immune system through CLEC7Aa C-type lectin receptor recognizing fungal wall β-glucans. **(B)** In IBD patient intestine, bacterial dysbiosis may contribute to increased methanogen species abundance, known to likely promote TNF production and activate dendritic cells (DCs), eventually contributing to the inflammatory state of the mucosa. *Candida kefyr* acts as a probiotic during intestinal inflammation by re-establishing the *Bacteroides* and *Lactobacillales* abundances. *S. cerevisiae* may exert anti-inflammatory effects by stimulating the IL10-production by DCs. This image has been designed using resources from https://www.twinkl.fr/ and https://www.flaticon.com/.

Detailed analysis of gut archaeome revealed the predominance in the intestine of *Methanobrevibacter (M.) smithii* and *Methanosphaera (M.) stadtmanae* species, belonging to the archaeal *phylum* of *Euryarchaeota* (Conway de Macario and Macario, 2009; Dridi et al., 2009; Gaci et al., 2014) and likely harboring the strong capability to establish a syntrophic association with several bacterial species (Samuel et al., 2007). Besides these two species, haloarchaea, a non-methanogenic euryarchaeota belonging to the salt-loving family of archaea (Kim et al., 2020) (Figure 1A), and other members of several archaeal orders have been identified as components of the gut microbiota. Among these, methanogenic members of the orders *Methanosarcinales*, *Methanobacteriales*, *Methanococcales, Methanomicrobiales*, and *Methanopyrales* were found to populate the human gut, along with the members of *Desulfurococcales, Sulfolobales, Thermoproteales, Nitrososphaerales*, and *Halobacteriales* orders, also detected in the human intestine (Gaci et al., 2014). A recent work, while confirming an increased prevalence of *M. stadtmanae* in the majority of human samples, failed to detect any non-methanogenic archaeal lineages (Raymann et al., 2017), unless the *Haloferax massiliensis* and *Haloferax assiliense*, demonstrating that halophilic archaea can inhabit the human gut (Khelaifia and Raoult, 2016; Khelaifia et al., 2018).

The contribution of the archaeome to the host physiology has not been completely understood, even if some relationship with the metabolism of dietary food has been highlighted, as for the *M. smithii*, found to trigger calories intake from the diet (Gaci et al., 2014). Moreover, methanogens exist in a syntrophic relationship with bacterial species within the gut. Indeed, short-chain fatty acids (SCFAs) such as acetate, propionate, and butyrate, as well as hydrogen gas, are produced as a result of anaerobic bacterial fermentation (Samuel and Gordon, 2006). As mentioned above, by eliminating H_2_ in the colon and thus affecting bacterial energy production, methanogens optimize the energy yield of the entire human microbiota (Chaudhary et al., 2018) (Figure 1A). As another example of host-microbiome interaction perpetuated by archaeal commensals, *Methanomassiliicoccus luminyensis*, recently isolated from human feces (Borrel et al., 2014), was found as sensitive to human-derived antimicrobial peptides and exhibited low immunogenicity toward human immune cells *in vitro*, thus perfectly resembling a commensal gut microbe (Bang et al., 2017).

Despite the evidence depicting the archaea as commensals, their role in IBD pathogenesis has not been fully elucidated yet (Aldars-García et al., 2021). Some indications come from studies regarding the *M. stadtmanae*, found to promote *in vitro* production of TNF and to be more abundant in IBD patient stools by comparison with the controls, suggesting a possible involvement in gut inflammation (Lecours et al., 2014) (Figure 1B). Importantly, *M. stadtmanae* was displayed to significantly activate human dendritic cells (Bang et al., 2014), further supporting this archaeon to participate in IBD pathogenesis (Lecours et al., 2014). Also, a recent report showed significantly lower *M. smithii* levels among IBD patients compared to healthy individuals, while normal *M. smithii* level was recovered in disease remission (Ghavami et al., 2018).

The recent IBD metatranscriptomics meta-analysis, the IBD TaMMA, analyzing a very large sample size (Massimino et al., 2021), highlighted for the first time differences in archaeome composition between colon and ileum from UC and CD patients. Indeed, such a transcriptome analysis compendium revealed colonic and ileal samples to largely differ in terms of archaeal compositions (Massimino et al., 2021). More interestingly, *Nitrosophaerales*, *Haloferacales*, *Natrialbales*, and *Thermococcales* were among the most abundant archaea orders in CD ileum, whereas the most abundant orders in UC ileum were *Methanococcales*, *Methanobacteriales*, *Methanosarcinales*, *Methanomicrobiales*, evidencing the differences between the two diseases in the ileal part, more “methanogenic” in UC. Also, *Methanomicrobiales* were found higher in UC colons, where it was the sole archaeal order to be statistically significant, while no differences were observed in colons from CD patients. From these insights, we can conclude that each intestinal tract may display differential abundances of archaea not only featuring the specific gut tract, but also the specific disease conditions. Such differences may contribute also to fostering diversified metabolic processes in the different intestinal segments, finally influencing the overall intestinal homeostasis. The archeaome composition in healthy conditions and the changes occurrgin during IBD pathogenesis are summarized in Table 1.

**TABLE 1:** Microbiota composition in healthy and IBD conditions.

**Kingdom**	**Phylum**	**Order**	**Genus/Species**	**IBD**	**References**
*Archaea*	*Crenarchaeota*	*Desulfurococcales*			Gaci et al., 2014
		*Sulfolobales*			Gaci et al., 2014
		*Thermoproteales*			Gaci et al., 2014
	*Euryarchaeota*	*Halobacteriales*	*Haloferax assiliense*		Gaci et al., 2014; Khelaifia and Raoult, 2016; Khelaifia et al., 2018; Kim et al., 2020
			*Haloferax massiliensis*		Gaci et al., 2014; Khelaifia and Raoult, 2016; Khelaifia et al., 2018; Kim et al., 2020
		*Haloferacales*		Increased	Massimino et al., 2021
		*Methanobacteriales*	*Methanobrevibacter smithii*	Decreased	Conway de Macario and Macario, 2009; Dridi et al., 2009; Gaci et al., 2014; Ghavami et al., 2018
			*Methanosphaera stadtmanae*	Increased	Conway de Macario and Macario, 2009; Dridi et al., 2009; Bang et al., 2014; Gaci et al., 2014; Lecours et al., 2014; Raymann et al., 2017
				Increased	Gaci et al., 2014; Massimino et al., 2021
		*Methanococcales*		Increased	Gaci et al., 2014; Massimino et al., 2021
		*Methanomassiliicoccales*	*Methanomassiliicoccus luminyensis*		Borrel et al., 2014
		*Methanomicrobiales*		Increased	Gaci et al., 2014; Massimino et al., 2021
		*Methanopyrales*			Gaci et al., 2014
		*Methanosarcinales*		Increased	Gaci et al., 2014; Massimino et al., 2021
		*Natrialbales*		Increased	Massimino et al., 2021
		*Thermococcales*		Increased	Massimino et al., 2021
	*Thaumarchaeota*	*Nitrososphaerales*		Increased	Gaci et al., 2014; Massimino et al., 2021
*Fungi*	*Ascomycota*	*Capnodiales*	*Cladosporium*		Qiu et al., 2020; Sokol et al., 2017
		*Eurotiales*	*Aspergillus clavatus*	Increased	Liguori et al., 2016; Massimino et al., 2021; Qiu et al., 2020; Sokol et al., 2017
			*Penicillium*	Increased	Massimino et al., 2021; Qiu et al., 2020; Sokol et al., 2017
		*Glomerellales*		Increased	Massimino et al., 2021
		*Hypocreales*	*Gibberella moniliformis*	Increased	Liguori et al., 2016; Qiu et al., 2020; Massimino et al., 2021;
		*Magnaporthales*		Increased	Massimino et al., 2021
		*Mycosphaerellales*		Increased	Massimino et al., 2021
		*Pleosporales*	*Alternaria brassicola*	Increased	Liguori et al., 2016; Qiu et al., 2020
		*Saccharomycetales*	*Candida albicans*	Increased	Mason et al., 2012; Chehoud et al., 2015; Sokol et al., 2017; Qiu et al., 2020; Massimino et al., 2021
			*Candida glabrata*	Increased	Hoarau et al., 2016; Liguori et al., 2016; Sokol et al., 2017; Qiu et al., 2020; Massimino et al., 2021
			*Candida tropicalis*	Increased	Hoarau et al., 2016; Liguori et al., 2016; Sokol et al., 2017; Qiu et al., 2020; Massimino et al., 2021
			*Debaryomyces*	Increased	Sokol et al., 2017; Qiu et al., 2020; Massimino et al., 2021
			*Galactomyces*	Increased	Sokol et al., 2017; Qiu et al., 2020; Massimino et al., 2021
			*Saccharomyces cerevisiae*	Decreased	Quinton et al., 1998; Chehoud et al., 2015; Sokol et al., 2017; Qiu et al., 2020; Massimino et al., 2021
		*Schizosaccharomycetales*		Decreased	Massimino et al., 2021
		*Sordariales*		Increased	Massimino et al., 2021
	*Basidiomycota*	*Malasseziales*	*Malassezia restricta*	Increased	Sokol et al., 2017; Qiu et al., 2020; Massimino et al., 2021
			*Malassezia sympodialis*	Decreased	Hoarau et al., 2016; Sokol et al., 2017; Qiu et al., 2020; Massimino et al., 2021
		*Tremellales*	*Cryptococcus*	Increased	Sokol et al., 2017; Qiu et al., 2020; Massimino et al., 2021
			*Cystofilobasidium*	Increased	Liguori et al., 2016; Qiu et al., 2020; Massimino et al., 2021;
			*Trichosporon*	Increased	Sokol et al., 2017; Qiu et al., 2020; Massimino et al., 2021
		*Ustilaginales*		Increased	Massimino et al., 2021
	*Mortierellomycota*				Qiu et al., 2020

*Archaeome and mycobiome species composing the gut microbiota independently of inflammatory conditions are indicated in green. Archaeome and mycobiome species shifts during IBD pathogenesis are indicated in blue and red for the downregulation and upregulation of these microbial entities, respectively.*

Notably, the IBD-associated bacterial dysbiosis may contribute to the archaeome composition shift, with an advantage of methylotrophic archaeal species (Figure 1B), particularly *M. stadtmanae*, finally increasing the inflammatory response within the human gut (Lecours et al., 2014). In this regard, a proposed leading theory explaining the possible contribution of archaea to IBD pathogenesis is that the SCFA butyric acid, produced in the colon by bacterial fermentation of dietary fibers and resistant starch, is a key regulator of syntrophism between archaea and bacteria in the gut (Matijašić et al., 2020). However, the archaeal overgrowth and the subsequent increased removal of SCFA from the biofilms are the two potential factors that cause dysbiosis, triggering bacteria to become endoparasites and enter intestinal epithelial tissues, which in turn leads to inflammatory processes in the human gut (White, 2017) (Figure 1B).

In conclusion, even if a causal link between archaeome dysbiosis and IBD pathogenesis has not been demonstrated yet, the evidence coming from previous studies suggested that archaea may play a major role in both health and IBD conditions. However, the way to the full definition of archaea-triggered mechanisms and functions in the gut is still long and deserves much more attention as a key player in regulating intestinal physiology. Moreover, its dysregulation may contribute, in combination with the other commensals, to sustain gut chronic inflammation, thus resulting important for the better comprehension of IBD pathogenesis and the development of new therapeutic lines of interventions aimed at the reconstitution of the archaeal composition within the intestinal microbiota.

### The Gut Mycobiome

Mycobiome is a general designation used for the description of fungal communities (molds and yeasts) of the microbiome (Cui et al., 2013) and is supposed to include nearly 0.1% of the total microbes in the gut (Arumugam et al., 2011; Nash et al., 2017). Mycobiome has been detected in the GI tracts of several mammals including humans, mice, rats, pigs, and many ruminants and non-ruminants (Liggenstoffer et al., 2010; Iliev et al., 2012).

Although most of the fungi-related studies were based on culture-dependent methods (Anderson, 1917), thus hampering the understanding of the fungal community of the microbiota, the recent advance in sequencing techniques (Meyer et al., 2010) has been progressively enlarging our knowledge about the mycobiome composition within the gut (Sokol et al., 2017; Qiu et al., 2020), where a lower diversity of fungal community than the bacterial was found (Nash et al., 2017).

In the human gut, many studies reported *Ascomycota, Basidiomycota*, and *Mortierellomycota* as the most dominant *phyla* of fungi (Qiu et al., 2020), while the most abundant *genera* composing the gut mycobiome are the *Candida* (particularly *C. albicans*), *Saccharomyces* (particularly *S. cerevisiae*), *Penicillium*, *Aspergillus*, *Cryptococcus*, *Malassezia* (particularly *M. restricta*), *Cladosporium*, *Galactomyces*, *Debaryomyces*, and *Trichosporon* (Sokol et al., 2017; Qiu et al., 2020) (Figure 1A).

Also, mycobiome entities exist in a tight equilibrium with the host and with the other actors of the intestinal microbiota, such as bacteria, thus altogether contributing to the maintenance of the overall tissue homeostasis (Santus et al., 2021). Evidence from experimental models showed *C. Albicans* to contribute to the recolonization of the intestine by bacterial species (*Bacteroides)* after antibiotic treatment (Mason et al., 2012). Interestingly, another study suggested the overall fungal and bacterial composition of the microbiota to be impacted by dietary habits in Japanese and Indian individuals. Results from this study revealed the higher abundance in Indian participants of *Candida* and *Prevotella*, maybe resulting from a higher dietary intake of vegetables used as a growth factor by the various *Candida* species (Pareek et al., 2019).

Some species populating the gut mycobiome were demonstrated to act as probiotics, with therapeutic potential for the host. As an example, the probiotic *Saccharomyces (S.) boulardii* was found to prevent antibiotic-associated diarrhea (Szajewska and Kołodziej, 2015) by regulating the immune system and by exerting an antimicrobial activity (Kelesidis and Pothoulakis, 2012). Another probiotic species is the *Candida kefyr*, found to reduce the severity of colitis by decreasing the abundance of *Bacteroides* and increasing the *Lactobacillales* (Takata et al., 2015) (Figure 1B). This shift in microbiota composition was associated with a decrease in interleukin (IL) 6 production (Takata et al., 2015). Moreover, it is suggested that fungi can be detected by the host immune system through Dectin-1 (CLEC7A), a c-type lectin receptor recognizing fungal wall β-glucans, resulting in a host immune response (Iliev et al., 2012) (Figure 1A).

Besides this beneficial impact on the host’s health, mycobiome dysbiosis was also associated with IBD. Higher levels of anti-*S. cerevisiae* antibodies raised against a component of the fungal cell wall were detected in CD patients’ sera compared to controls resulting in a reliable CD biomarker and predictors of the disease course (Quinton et al., 1998).

Reduced fungal diversity and increased abundance of *Candida* (*C.*) species were found in both pediatric and adult IBD patients (Figure 1B). More specifically, in adult IBD patients an increased *Basidiomycota/Ascomycota ratio* was found, coupled with the enrichment of *C. albicans* and the reduction of *S. cerevisiae* (Chehoud et al., 2015; Sokol et al., 2017). Moreover, *Gibberella Moniliformis*, *Alternaria Brassicola, Aspergillus clavatus*, and the *Cystofilobasidiaceae* were found to be increased in IBD patients (Liguori et al., 2016), while *Malassezia sympodialis* was markedly decreased (Hoarau et al., 2016). In CD, *C. cropicalis* and *C. glabrata* were found augmented by comparison with the control (Hoarau et al., 2016; Liguori et al., 2016).

Fungal dysbiosis observed in IBD patients was found to be associated also with composition shifts of other microbial compartments. As an example, the reduction of *S. cerevisiae* was observed in association with the reduction of several bacterial genera, such as *Bifidobacterium*, *Blautia*, *Roseburia*, and *Ruminococcus* (Qiu et al., 2020).

From a mechanistic point of view, the intestinal mycobiome was delineated also as a contributor during the inflammatory process. For example, by treating bone marrow-derived dendritic cells with two heat-killed yeast strains of *S. cerevisiae* and *C. albicans*, Sokol and colleagues found that the IL6 levels were comparable among treatments, but the anti-inflammatory cytokine IL10 was significantly higher after the stimulation with *S. cerevisiae* by comparison with the *C. albicans* (Figure 1B), suggesting an anti-inflammatory effect of the former (Sokol et al., 2017).

Additionally, *C. tropicalis* together with *E. coli* and *Serratia marcescens* bacteria were found to form a biofilm functioning as a commensal niche enriched in fungal hyphae, usually increased in pathogenic conditions (Hoarau et al., 2016). Interestingly, *C. tropicalis* was shown to positively correlate with *Serratia marcescens* and *Escherichia coli* in CD, further supporting their role in sustaining chronic inflammation as a “team” in the commensal niche (Hoarau et al., 2016).

The widely accepted mechanisms through which intestinal mycobiota may interact with the host’s mucosal physiology are prevalently based on the interaction between fungal species and innate immunity, involving specific receptors and signals driving the inflammatory response (Mahmoudi et al., 2021).

Among the main types of innate immune receptors that can recognize fungal Pathogen-Associated Molecular Patterns (PAMPs), Toll-Like Receptors (TLRs), C-type lectin receptors (CLRs), NOD-like receptors (NLRs), and galectin 3 on antigen-presenting cells are well recognized and characterized (Bourgeois and Kuchler, 2012). The most investigated belonging to the CLRs include Dectin-1, recognizing the PAMP β-glucan (Taylor et al., 2007), Dectin-2, Dendritic cell-specific intercellular adhesion molecule-3-grabbing non-integrin receptor (DC-SIGN), Macrophage inducible Ca^2+^-dependent lectin receptor (MINCLE), suggested to recognize the α-mannose (Yamasaki et al., 2009), the Mannose Receptor (MR). Some of CLRs were found to directly interact with TLRs to recognize fungi (Vautier et al., 2012). Within the intestine, also, the fractalkine receptor (CX3CR-1) expressed by resident mononuclear phagocytes was recently discovered to mediate the interaction between the intestinal mycobiota and host immunity in both health conditions and inflammatory state (Leonardi et al., 2018). The proposed mechanism through which fungal recognition by immune cells triggers an innate immunity cascade occurs through the activation of the spleen tyrosine kinase (SYK) or the SYK-independent RAF-1 activation, which ultimately trigger the NF-κB signaling pathway toward T helper 1 and/or T helper 17 immunophenotypes (Richard and Sokol, 2019).

Susceptibility against mycobiome-related complications in IBD patients may rely on the genetic alteration within fungal recognition receptors-encoding genes. As some examples, polymorphisms in genes encoding for *Detectin-1, TLR-1 and 3, MINCLE, Caspase recruitment domain-containing protein 9 (CARD9), Dectin-1, CD209, and CX3CR1* are the most investigated as associated with fungal dysbiosis/altered innate immunity during IBD pathogenesis (Iliev et al., 2012; Sokol et al., 2017; Leonardi et al., 2018; Limon et al., 2019).

The majority of the studies reported so far elucidated the mycobiome composition in small sample-sized IBD patient cohorts, mainly on stools and exploiting targeted DNA sequencing and PCR (Mahmoudi et al., 2021). By contrast, IBD TaMMA (Massimino et al., 2021) highlighted CD and UC ilea both featuring an increased abundance of *Glomerellales*, *Tremellales*, and *Hypocreales*, coupled with decreased abundance of *Schizosaccharomycetales*. Some orders were instead differentially abundant in the two conditions. *Saccharomycetales*, *Ustilaginales*, *Malasseziales*, *Eurotiales*, *Mycosphaerellales*, and *Magnaporthales* were found to be differentially abundant exclusively in UC ileum, while *Saccharomycetales*, *Ustilaginales*, and *Sordariales* were dysregulated only in CD ileum. Interestingly, differently from the ileal tract, very few orders were found to be differentially abundant in colons, perhaps resembling the different immune competence of the two tissues (Mann et al., 2016).

The mycobiome composition in healthy conditions and during IBD pathogenesis are summarized in Table 1.

Besides the long list of results coming from experimental colitis models extensively narrated by Beheshti-Maal and colleagues (Beheshti-Maal et al., 2021) and discussed later in this review, the real link between IBD pathogenesis and mycobiome dysbiosis is still missing, although some evidence lied ahead. Indeed, by combining the results from previous studies, fungal dysbiosis has been emerging to likely modulate the IBD bacteriome (Iliev and Leonardi, 2017), known to be pivotal in chronic inflammation onset and perpetuation as much as the immune system (Iliev and Leonardi, 2017). In this regard, in a study comparing samples from CD patients and their not-affected relatives, the inter- and intra-kingdom interactions between bacteriome and mycobiome were proposed to impact the host’s immune system in the setting of CD. Specifically, *C. tropicalis* interacts with potential bacterial pathogens, and that these interactions may play an important role in CD pathogenesis (Hoarau et al., 2016). Of note, the authors also indicated the microbiotas, specifically the mycobiomes, of familial samples as distinct from the non-familial (Hoarau et al., 2016). This may be partially explained by the consensus that members of a family share genetics, environment, diet, and bacterial microbiota being more similar to each other than they are to the unrelated individuals (Schloss et al., 2014).

Additionally, mycobiome entities were proposed to directly intervene in the release of IBD-specific proinflammatory cytokines (Beheshti-Maal et al., 2021), as suggested by the correlation found between specific gut mycobiome compositions with the expression levels of a series of pro-inflammatory cytokines in UC inflamed mucosa (Qiu et al., 2017). As a consequence, it is reasonable to indicate the mycobiome composition alterations, such as the increased abundances of *Candida* species often found in IBD mucosa, to be associated with inflammation and disease severity, as previously shown by Li and colleagues (Li et al., 2014). Consistently, Kowalska-Duplaga and colleagues observed that the abundance of *Candida* in CD patients decreased during the therapeutic intervention, particularly with anti-TNFα treatment (Kowalska-Duplaga et al., 2019), indicating a possible direct link between pro-inflammatory conditions and mycobiome composition. Besides the IBD-associated *Candida* species enrichment, an unbalanced *Basidiomycota/Ascomycota* ratio was also observed to correlate with flare-up/remission conditions in IBD (Sokol et al., 2017).

Overall, these pieces of evidence, although not specifically defining the mycobiome-triggered functional mechanisms underlying IBD pathogenesis, propose the mycobiome as important as the other microbiota compartments in orchestrating and contributing to the chronic gut inflammation onset and perpetuation, thus opening additional horizons for the investigation of IBD-associated microbiota diversity.

## Major Challenges in Studying Mycobiome and Archeome

Even if archaea resemble bacteria as we discussed above, they are so evolutionarily distant from bacteria that they retain some eukaryotic traits (i.e., molecular machinery for transmission and manipulation of genetic information) (Borrel et al., 2020). Such diversity makes challenging their detection and analysis if bacteria-centric methodologies are exploited, such as the nucleic-acid-based fluorescence *in situ* hybridization, cultivation, and molecular quantitative analyses. In the majority of commercially available kits for DNA extraction, for example, lysozyme is one of the most-used components. However, this is not suitable for archaeal DNA extraction since it cannot disrupt archaeal pseudo-peptidoglycan (Borrel et al., 2020). Indeed, more aggressive treatments may be required for archaeal cell wall break, as in the case of *Methanobacteriales* (Lee et al., 2009). No fewer difficulties are encountered while performing molecular analysis, where the so-called “universal” 16S rRNA primers fail to cover the broad archaeal diversity and to correctly annotate certain archaeal lineages (Mahnert et al., 2018). This hurdle is even huger if we consider the limited availability of well-annotated genomes in under-represented archaeal *phyla*, eventually failing to correctly assign archaeal sequences (Mahnert et al., 2018).

Similar limitations are met when fungi should be analyzed. Although some fungal entities can be cultured *in vitro* (Chevalier et al., 2018) and used to manipulate animal models as we discussed later in this review, there are significantly few complete fungal genomes yet available (Underhill and Iliev, 2014), making also their classification challenging. The approach most commonly used for fungal analysis is to amplify the fungal “internal transcribed spacer” (ITS) regions (Tang et al., 2015). Since the ITS regions are not part of the conserved transcribed regions of the structural ribosomal RNAs, they are highly divergent between fungi, allowing their classification at the species level. However, fungal ITS sequences can differ widely in size and sequence content (Santamaria et al., 2012) and there is no well-established database of ITS sequences (Tang et al., 2015).

As a matter of fact, in both mycobiome and archaeome contexts, the use of metatranscriptomics may help to better classify and annotate the fungal and archaeal composition of the gut microbiota, as we recently reported in IBD TaMMA (Massimino et al., 2021). However, much more effort in developing and improving both sequencing and cultivation approaches is required to make plausible the mycobiome and archaeome studies to unravel their physiological properties. This also represents a major limitation that renders the animal and human studies for elucidating their role during intestinal inflammation harder, as we discuss shortly.

## The Role of Mycobiome and Archaeome During Intestinal Inflammation: Evidence From Animal Studies

### The Mycobiome in Experimental Colitis

Whether on one hand human studies are relevant from a clinical point of view, unfortunately, they cannot provide mechanistic and functional notions that may help to determine whether gut mycobiota and archaeome dysbiosis are causal for intestinal inflammation onset and progression or this is only a consequence associated with the inflammatory process. Over the years, the established models of experimental colitis have been offering the chance to depict a more comprehensive view of the entire process involving intestinal microbiota in the aetiogenesis of gut inflammation, mainly illustrating the intestinal bacteriome roles (Zhang et al., 2017) and, to a lesser extent, the mycobiome and archaeome functions. As an example, Qiu and colleagues reported how the fungal composition of the GI tract may change during Dextran sulfate sodium (DSS)-induced colitis, finally uncovering that, while fungi were higher in the ileal tract by comparison with the colon independently of the inflammation state, the fungal Shannon diversity index of the DSS-induced colitis mice was lower than the controls in each gut segment (Qiu et al., 2015). Additionally, the inflammatory process caused a fungal translocation from the gut lumen to extra-enteric organs (such as the spleen and the mesenteric lymph node) during the experimental model of chronic colitis only in the inflamed intestinal tracts (Qiu et al., 2015), indicating that the inflammatory process itself may accelerate distal fungal invasion, likely because of the increased intestinal permeability. In line with previous evidence, the authors also showed that the fungal depletion was paralleled with a shift in mucosal bacterial composition (Qiu et al., 2015).

The direct association between fungi and bacteria during experimental intestinal inflammation was shown in another recent study highlighting *Enterobacteriaceae* to have a positive effect on fungal colonization of the gut, finally influencing the progression of gut inflammation. Indeed, *C. albicans* requires the presence of specific bacteria that trigger intestinal inflammation to increase the intensity, so that antibiotic treatment resulted as beneficial against DSS-induced colitis (Sovran et al., 2018). Also, *C. albicans* colonization of mouse intestines induced a strong Th17 response, suggesting that fungal composition infer specific immune changes in the host. Furthermore, Chiaro and colleagues in 2017 demonstrated that increased intestinal colonization with S. cerevisiae aggravated colitis by influencing purine metabolism, leading to extensive damage of the gut epithelia, reversible thanks to the inhibition of the purine pathway (Chiaro et al., 2017). These results further strengthened the concept that a tight interaction between mycobiota and the host’s metabolism exists, overall influencing the host’s physiology.

### The Archaea in Experimental Colitis

While some studies about fungi are existing in the context of experimental colitis, fewer pieces of evidence described the functional contribution by archaea during gut inflammation *in vivo*. This is mainly due to the difficulty in isolating archaeal species (Vemuri et al., 2020), and thus the functional studies *in vivo* manipulating the archaeome composition are still missing. Nevertheless, a very recent study reported its characterization in animals. Indeed, Mohamed and colleagues found methanogens to be increased during experimental colitis, coupled with bacterial dysbiosis, thus confirming the importance of archaeome-bacteriome equilibrium also *in vivo* (Mohamed et al., 2021).

Moreover, previous evidence reported that *Methanobrevibacter smithii* contributed to digestive health by directing *Bacteroides thetaiotaomicron*-mediated fermentation of dietary fructans to acetate, and in turn *B. thetaiotaomicron*-derived formate fostered *M. smithii* for methanogenesis, thus demonstrating a link between this archaeon and bacterial utilization in balancing host’s metabolism (Samuel and Gordon, 2006). Although this study did not demonstrate the effect of methanogens during intestinal inflammation, it does propose their contribution to metabolic health, suggesting their involvement in maintaining tissue physiology.

Conclusively, the paucity of studies about mycobiome and archaeome manipulation *in vivo* during intestinal inflammation strongly suggests that there is an urgent need to enlarge the knowledge about the mechanism directed by these two neglected components of the intestinal microbiota to make a step forward to the full comprehension of the entire microbiota-mediated mechanism in health and disease.

## Manipulating the Gut Mycobiome and Archaeome for the Treatment of Inflammatory Bowel Disease: Evidence From Clinical Trials

Alteration in gut microbiota compositions during IBD pathogenesis and the evidence that specific microbial entities may result beneficial, or detrimental, have been leading over the years to the development of an enormous number of clinical trials, as those assessing the efficacy of the Fecal Microbiota Transplant (FMT) (Oka and Sartor, 2020). Reasonably, FMT exerts beneficial effects by transferring fecal microbiota from a healthy individual to an IBD patient, re-establishing the correct balance among microbial entities in the gut. Also, antibiotics are used as primary therapy for inducing or maintaining remission based on the hypothesis that certain bacteria cause IBD (Sartor and Wu, 2017). Likewise, virome modulation has been proposed as beneficial by administering CD patients with a bacteriophage cocktail parasitizing *adhesive-invasive E. coli*. Currently, this approach is under investigation in a clinical trial (NCT03808103) (Ungaro et al., 2019a).

Regarding the modulation of fungal composition, treatments may encompass some anti-fungal medications. Specifically, the NCT03476317 small pilot study has completed the recruitment of patients to determine the effect of a novel gut microbiota-targeted therapeutic regimen (bowel lavage and antibiotics with or without the antifungal fluconazole) in the management of active CD or indeterminate colitis (IBDU) that is refractory to conventional, immunosuppressive therapy.

Among future clinical trials, the NCT05049525, not recruiting yet, aims at the evaluation of the response of the combined anti-fungal itraconazole and terbinafine therapies compared to placebo in patients with CD, further strengthening the concept that targeting fungal entities in these patients may help their remission. Similarly, the NCT04966585 pilot study, not recruiting yet, will investigate whether the microbial changes induced by antifungal treatment are associated with dampened downstream immune responses in CD patients with a genetic predisposition to developing strong immune responses to *Malassezia*.

It is noteworthy that the use of probiotics, including live biotherapeutic products (LBPs) based on bacterial- and fungal-derived molecules, is establishing a new line of treatment of IBD patients (Oka and Sartor, 2020). In specific regard to fungal-derived factors, a randomized clinical trial assessing the efficacy of *Saccharomyces boulardii*, Plein and colleagues observed an improved disease activity index in a cohort of CD patients (Plein and Hotz, 1993). Similar trials have been performed later, in CD patients, highlighting improved relapse rate (Guslandi et al., 2000) and intestinal permeability (Garcia Vilela et al., 2008).

The possibility to target archaeome is more challenging. Indeed, human methanogenic archaea are highly resistant to antibiotics (Dridi et al., 2011), being susceptible only to molecules that are also effective against both bacteria and eukarya, thus hampering a possible specific therapy. Quite recently, however, statins have been elucidated as inhibitors of archaeal cell membrane biosynthesis without affecting bacterial numbers, opening the possibility of a therapeutic intervention that targets a specific aetiological factor while protecting the intestinal microbiome (Gottlieb et al., 2016). This may be the starting point also for the modulation of the archaeome in IBD patients as a therapeutic intervention, even if the route to success is still long and much more effort and attention to this aspect for IBD treatment need to be dedicated.

## Concluding Remarks

Inflammatory Bowel Disease (IBD) is a complex disease where different factors, ranging from cytokines, molecules, to immune cells and microbial entities, play major roles in directing and sustaining chronic inflammation. Despite the enormous number of studies aimed and the definition of its aetiogenesis, at present, IBD is dominated by repetitive technology-based analyses of gut dysbiosis and by clinical trials based on the cyclical blockade of an endless series of cytokines, signaling molecules, and homing receptors. It is evident that the field of IBD currently lacks fresh concepts and original discoveries. Hence, more NGS-based investigations for the elucidation of the mechanisms sustained by intestinal microbiota components are urgently needed to lay down new hypotheses and raise theories delineating the IBD aetiopathogenesis. At the same time, more animal studies are required to further elucidate functions and mechanisms mediated by all components of the intestinal microbiota, including the more neglected such as fungi and archaea.

In this direction, the full comprehension of all microbiota components that may cause IBD onset and progression will help to develop novel therapeutic strategies that will finally consider IBD in its real nature, that is complexity and heterogeneity. Establishing how the diverse microbial commensals interact with each other and with the host is the basis for solving this complexity, finally leading to tailored therapies considering patient-specific characteristics within the intestinal microbiota.

## Author Contributions

YH, LM, LL, and FU: conceptualization and writing, review, and editing. SD and FU: supervision, review, and editing. FU: funding acquisition. All authors contributed to the article and approved the submitted version.

## Conflict of Interest

SD has served as a speaker, consultant, and advisory board member for Schering Plough, Abbott (AbbVie) Laboratories, Merck and Co, UCB Pharma, Ferring, Cellerix, Millenium Takeda, Nycomed, Pharmacosmos, Actelion, Alfa Wasserman, Genentech, Grunenthal, Pfizer, AstraZeneca, Novo Nordisk, Vifor, and Johnson and Johnson. The remaining authors declare that the research was conducted in the absence of any commercial or financial relationships that could be construed as a potential conflict of interest.

## Publisher’s Note

All claims expressed in this article are solely those of the authors and do not necessarily represent those of their affiliated organizations, or those of the publisher, the editors and the reviewers. Any product that may be evaluated in this article, or claim that may be made by its manufacturer, is not guaranteed or endorsed by the publisher.
